# P-2323. Utilizing Nanopore Sequencing Technology to Identify Saporvirus Genotypes in Fecal Samples from Young Children with Diarrhea in Al Ain City, United Arab Emirates

**DOI:** 10.1093/ofid/ofae631.2475

**Published:** 2025-01-29

**Authors:** Ahmed R Alsuwaidi, Junu George, Mahmoud Abdelrahman, Abdulrahman Alblooshi

**Affiliations:** United Arab Emirates University, Al Ain, Abu Dhabi, United Arab Emirates; United Arab Emirates University, Al Ain, Abu Dhabi, United Arab Emirates; United Arab Emirates University, Al Ain, Abu Dhabi, United Arab Emirates; United Arab Emirates University, Al Ain, Abu Dhabi, United Arab Emirates

## Abstract

**Background:**

Human sapoviruses are important pathogens causing diarrhea globally. They are increasingly detected in individuals with diarrhea due to the expanded utilization of molecular diagnostic methods. There are 18 genotypes known to infect human (GI.1-7, GII.1-8, GIV.1, and GV.1-2). Notably, the genetic characteristics of sapoviruses circulating in the United Arab Emirates (UAE) remain unclear.

This study aims to use next generation sequencing to analyze sapovirus genotypes in stool samples from children under five with diarrhea in Al Ain city.

**Methods:**

Using multiplex one-step real-time PCR (Allplex™ Gastrointestinal Full Panel Assay), 15 sapovirus-positive stool samples were identified from stool specimens collected for a previous epidemiological study of pathogens causing diarrhea during the period from December 2017 and April 2019. After excluding isolates with low viral load (Ct >30), 8 isolates were used for genotyping studies. Total RNA was extracted using a stool total RNA isolation kit and confirmed by endpoint PCR. Viral RNA was retrotranscribed to cDNA, followed by viral genome amplification, purification, and quantification. After sequencing with Nanopore MinION technology, reads that passed quality control (score >7) were produced. Geneious Prime and Mega11 Software were then used for base-calling, mapping, and phylogenetic analysis.

**Results:**

PCR amplicon-based platform was developed for sequencing human sapoviruses in order to reduce the cost of sequencing per sample. The system produced successful amplification in 7 out of 8 studied sapovirus-positive samples. All isolates showed close similarity with each other showing more than 99% similarity. Blast analysis of sequences of the amplicons revealed close similarity with East Asian isolates (99%). The closest Middle Eastern isolate was from Iran showing 87% similarity with the UAE isolates. Phylogenetic analysis revealed that seven of the eight sapovirus isolates were found to cluster within the GI.1 genotype.

**Conclusion:**

Using Nanopore sequencing technology, GI.1 genotype was found to be the most prevalent sapovirus genotype among the studied isolates. The obtained data will enhance our knowledge on the molecular diversity of sapoviruses in the UAE and enrich the current sapoviruses genotyping protocols.

**Disclosures:**

All Authors: No reported disclosures

